# Дилатационная кардиомиопатия у пациента с болезнью Кушинга — клиника, диагностика и лечение: описание случая

**DOI:** 10.14341/probl13147

**Published:** 2025-09-14

**Authors:** А. Б. Кузнецов, A. Ю. Григорьев, В. А. Кузнецов, Ж. Е. Белая, Л. Я. Рожинская

**Affiliations:** Национальный медицинский исследовательский центр эндокринологии им. академика И.И. Дедова; I.I. Dedov Endocrinology Research Centre; Первый медицинский государственный медицинский университет им. И.М. Сеченова (Сеченовский университет); Sechenov First Moscow State Medical University

**Keywords:** гипофизарная гиперсекреция АКТГ, болезнь Кушинга, дилатационная кардиомиопатия, сердечная недостаточность, описание случая, pituitary ACTH hypersecretion, Cushing disease, cardiomyopathy, dilated, heart failure, case report

## Abstract

Cortisol-induced dilated cardiomyopathy (CI-DCM) is a rare manifestation of endogenous hypercortisolism (EH). Optimal management of patients with CI-DCM is a major challenge due to the rarity of the pathology and the lack of expert community guidelines. This article describes a case of successful management of a patient with ACTH-secreting pituitary tumor and CI-DCM.

A 44-year-old patient was hospitalized with symptoms of chronic heart failure (CHF) and EH. The diagnosis of non-ischemic myocardial damage with phenotype of DCM was verified by echocardiography and coronary angiography. According to hormonal and imaging tests, and selective blood sampling from the inferior petrosal sinuses, an ACTH-secreting pituitary adenoma was diagnosed. A transnasal transsphenoidal adenomectomy was planned. Due to the symptoms of CHF and systolo-diastolic dysfunction of the left ventricle (LV), significantly increasing the risk of adverse perioperative cardiac events, the intervention was postponed. Stabilization of the patient’s condition was achieved after 4-month therapy with use of betaAB, ACEI, MRA, diuretics, and steroidogenesis inhibitors. Stabilization of the patient’s condition allowed to perform transnasal transsphenoidal adenomectomy without perioperative complications, with postoperative decrease of ACTH and cortisol levels. Follow-up examinations demonstrated preservation of eucorticism, regression of CHF symptoms. progressive decrease of LV size/volumes with increase of LVEF.

Cortisol hypersecretion can damage myocardium with a phenotype of DCM, with symptoms of CHF being the dominant clinical manifestation of EH. The use of betaAB, ACEI, diuretics, MRA, and steroidogenesis inhibitors is reasonable to control symptoms of CHF and prepare a patient with CI-DCM for surgical intervention. After normalization of cortisol level, regression of CHF symptoms and significant reduction of heart chamber size/volumes with increase of LVEF are noted, which allows to conclude about reversibility of pathologic cardiac remodeling.

Кортизол-индуцированная дилатационная кардиомиопатия (КИ-ДКМП) является достаточно редкой клинической манифестацией эндогенного гиперкортицизма (ЭГ) [1][2]. Диагностика и оптимальное ведение пациентов с КИ-ДКМП является серьезной мультидисциплинарной проблемой, что обусловлено редкостью данной патологии, отсутствием значимой доказательной базы и рекомендаций экспертных сообществ. Представлено описание клинического случая пациента с АКТГ-секретирующей опухолью гипофиза и КИ-ДКМП.

При описании случая авторы руководствовались рекомендациями CARE [3].

При измерениях и оценке ЭХОКГ-параметров авторы следовали текущим рекомендациям [4][5].

Пациент 44 лет был госпитализирован в ЭНЦ в июле 2017 г. с жалобами на одышку при незначительных физических нагрузках, выраженные периферические отеки, прогрессирующее увеличение массы тела, повышенную утомляемость.

В 2007 г., в возрасте 34 лет, отмечен дебют артериальной гипертензии (АГ). В последующие два года отмечалось появление и прогрессирование симптомов сердечной недостаточности (СН) в виде одышки и утомляемости при обычных физических нагрузках.

В 2012 г. диагностирована ДКМП, инициирована терапия бета-адреноблокаторами (бетаАБ), блокаторами рецепторов ангиотензина (БРА), дигидропиридиновыми блокаторами медленных кальциевых каналов (БМКК) и диуретиками (рис. 1).

**Figure:**
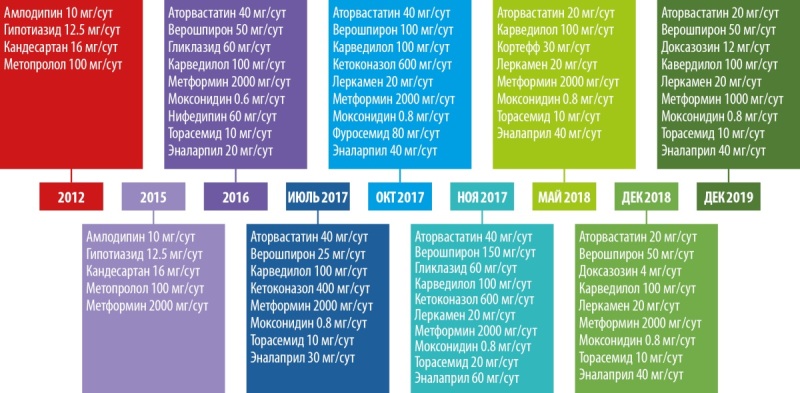
Рисунок 1. Модификация терапии по ходу лечения.

В 2015 г. в связи с прогрессированием симптомов СН и резистентностью к проводимой медикаментозной терапии была запланирована имплантация устройства для сердечной ресинхронизирующей терапии. При предоперационном обследовании было обращено внимание на прогрессирующее увеличение массы тела, выраженную мышечную слабость, плохое заживление кожных микротравм. Тогда же был диагностирован сахарный диабет (СД). Совокупность этих симптомов позволила заподозрить наличие ЭГ. К терапии был добавлен метформин (рис. 1).

В 2016 г. на основании проведенных гормональных тестов у пациента диагностирован АКТГ-зависимый гиперкортицизм, терапия была модифицирована (рис. 1).

При осмотре отмечено наличие ожирения III степени (рост 178 см, вес 142 кг, ИМТ 44,8 кг/м²) с относительно равномерным распределением подкожно-жировой клетчатки. На коже живота имелись неширокие (менее 1,5 см) розовые стрии. Умеренные отеки стоп и голеней. При аускультации легких хрипов не отмечалось, тоны сердца умеренно приглушены. АД — 160/90 мм рт.ст., пульс 80–100/мин, регулярный.

## ВРЕМЕННАЯ ПОСЛЕДОВАТЕЛЬНОСТЬ СОБЫТИЙ

Представлена на рисунке 2.

**Figure:**
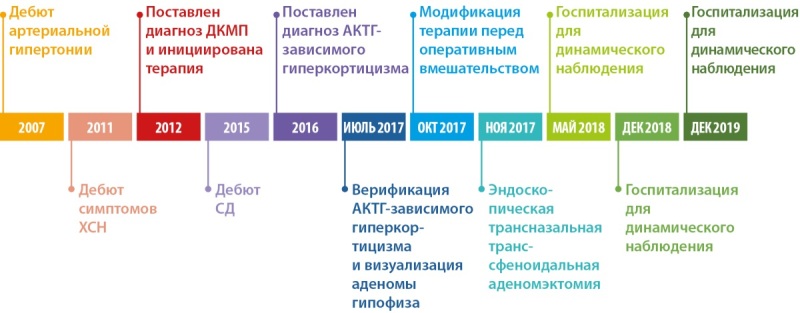
Рисунок 2. Временная последовательность событий.

## ДИАГНОСТИКА

Основной задачей первой госпитализации в ЭНЦ было подтверждение диагноза «ЭГ» и определение его типа. Применяемые гормональные тесты, их значения и референсные интервалы представлены в таблице 1. Результаты тестов позволили подтвердить наличие АКТГ-зависимого гиперкортицизма.

**Table:** Таблица 1. Показатели секреции кортизола и АКТГ

Тест/метод	Значение	Референсные значения
Предоперационные показатели
Кортизол (слюна, утро) (Cobas)	27,0 нмоль/л	6,8–25,9
Кортизол (слюна, вечер) (Cobas)	27,4 нмоль/л	0,5–9,4
Свободный кортизол мочи	507 нмоль/сут	60–413
АКТГ (плазма, утро)	79,91 пг/мл	7–66
АКТГ (плазма, вечер)	96,29 пг/мл	0–30
Постоперационные показатели
1-е сутки после операции
Кортизол (кровь, утро) (Cobas)	122 нмоль/л	123–626
АКТГ (утро)	8,76 пг/мл	7–66
6-е сутки после операции
Кортизол (кровь, утро) (Cobas)	21,66 нмоль/л	123–626
АКТГ (утро)	5,73 пг/мл	7–66
5 месяцев после операции (1-е контрольное исследование)
Кортизол (кровь, утро) (Cobas)	343,5 нмоль/л	123–626
АКТГ (утро)	28,32 пг/мл	7–66
12 месяцев после операции (после отмены Кортеффа; 2-ое контрольное исследование)
Кортизол (кровь, утро) (Cobas)	391,2 нмоль/л	123–626
Кортизол (кровь, вечер) (Cobas)	91,48 нмоль/л	46–270
Свободный кортизол мочи	117,6 нмоль/сут	60–413
24 месяца после операции (3-е контрольное исследование)
Кортизол (кровь, утро) (Cobas)	403 нмоль/л	171–536
Кортизол (кровь, вечер) (Cobas)	73,27 нмоль/л	64–327
Свободный кортизол мочи	203 нмоль/сут	100–379
АКТГ (утро)	44,51 пг/мл	7,2–63,3
АКТГ (вечер)	15,59 пг/мл	2–25,5

При госпитализации в июле 2017-го были предприняты попытки установить источник гиперсекреции АКТГ. МРТ головного мозга с контрастным усилением не позволила обнаружить визуализируемую аденому гипофиза. Однако селективный забор крови из нижних каменистых синусов выявил выраженный градиент АКТГ «центр-периферия», что позволило сделать вывод о гипофизарном источнике гиперсекреции и подтвердило диагноз болезни Иценко-Кушинга (БИК).

По данным МРТ выявлены дилатация левых камер сердца, гипертрофия ЛЖ, диффузный гипокинез ЛЖ со снижением ФВ ЛЖ до 30%, что соответствует клинико-морфологическому фенотипу ДКМП.

Пациент имел множественные факторы кардиоваскулярного риска (АГ, СД, атерогенная дислипидемия). С целью исключения ишемического генеза дисфункции ЛЖ была проведена коронароангиография (КАГ) (рис. 3). КАГ не выявила поражений коронарных артерий. Таким образом, было подтверждено предположение о неишемическом характере поражения миокарда.

**Figure:**
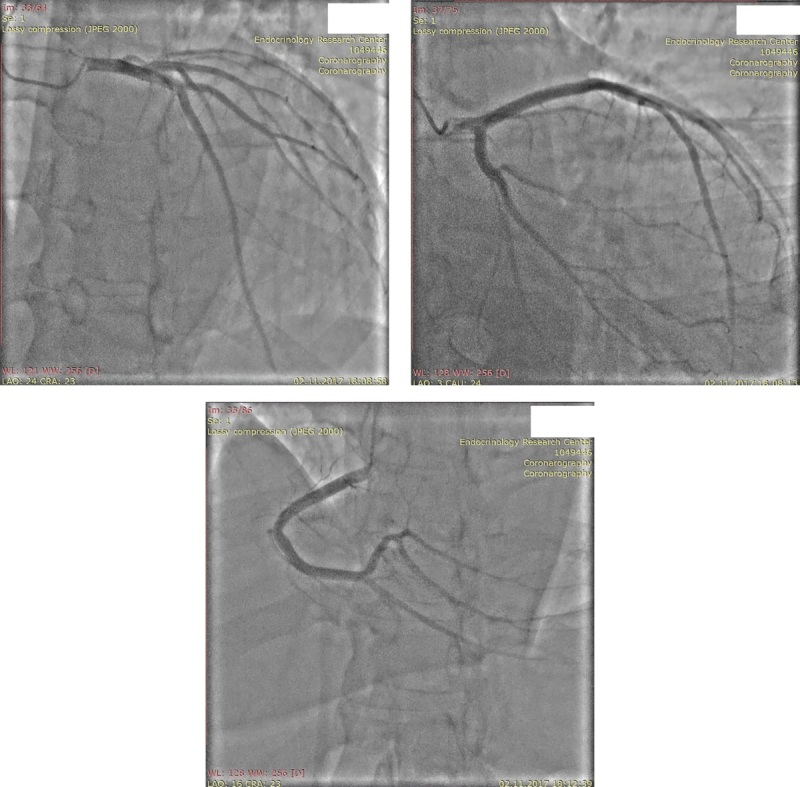
Рисунок 3. Коронароангиография. Примечание: сверху слева — ствол левой коронарной артерии, сверху справа — передняя межжелудочковая артерия, снизу — правая коронарная артерия.

## ЛЕЧЕНИЕ

По результатам обследования было принято решение о проведении эндоскопической трансназальной транссфеноидальной аденомэктомии.

Хроническая СН (ХСН) с толерантностью к нагрузкам на уровне III функционального класса по NYHA и признаками выраженной систолической дисфункции ЛЖ ассоциируется с высоким риском неблагоприятных периоперационных кардиальных и тромбоэмболических событий. Коллегиально было принято решение отложить оперативное вмешательство и стабилизировать состояние пациента. Проводимая терапия была модифицирована — увеличены дозы бетаАБ и ингибиторов ангиотензин-превращающего фермента (ИАПФ), добавлены моксонидин и кетоконазол (рис. 1).

При госпитализации в октябре 2017 г. для проведения оперативного вмешательства было отмечено прогрессирование симптомов ХСН: выраженное снижение толерантности к нагрузкам с развитием одышки при минимальной физической активности, ночное ортопноэ, выраженные периферические отеки, физикальные симптомы легочного застоя (влажные хрипы в нижних отделах легких), гепатомегалия. Пациент сообщил, что получал предписанную терапию в полном объеме. На ЭХОКГ были отмечены выраженная дилатация левых камер сердца, признаки гипертрофии ЛЖ, диффузный гипокинез ЛЖ с выраженным снижением ФВ ЛЖ (19%), рестриктивная диастолическая дисфункция ЛЖ (рис. 4; табл. 2). Таким образом, несмотря на проводимую терапию, отмечено прогрессирование симптомов ХСН с дальнейшим снижением ФВ ЛЖ и развитием выраженной диастолической дисфункции. Потребовалась модификация терапии: назначение внутривенных диуретиков (фуросемид 80 мг) в течение 5 дней. После внутривенной диуретической терапии исчезли эпизоды ночного ортопноэ и физикальная симптоматика легочного застоя, существенно уменьшились периферические отеки. Постоянная пероральная терапия была модифицирована: добавлены БМКК, а также увеличены дозы пероральных диуретиков, ИАПФ, бетаАБ и кетоконазола (рис. 1). Операция была отложена на один месяц.

**Figure:**
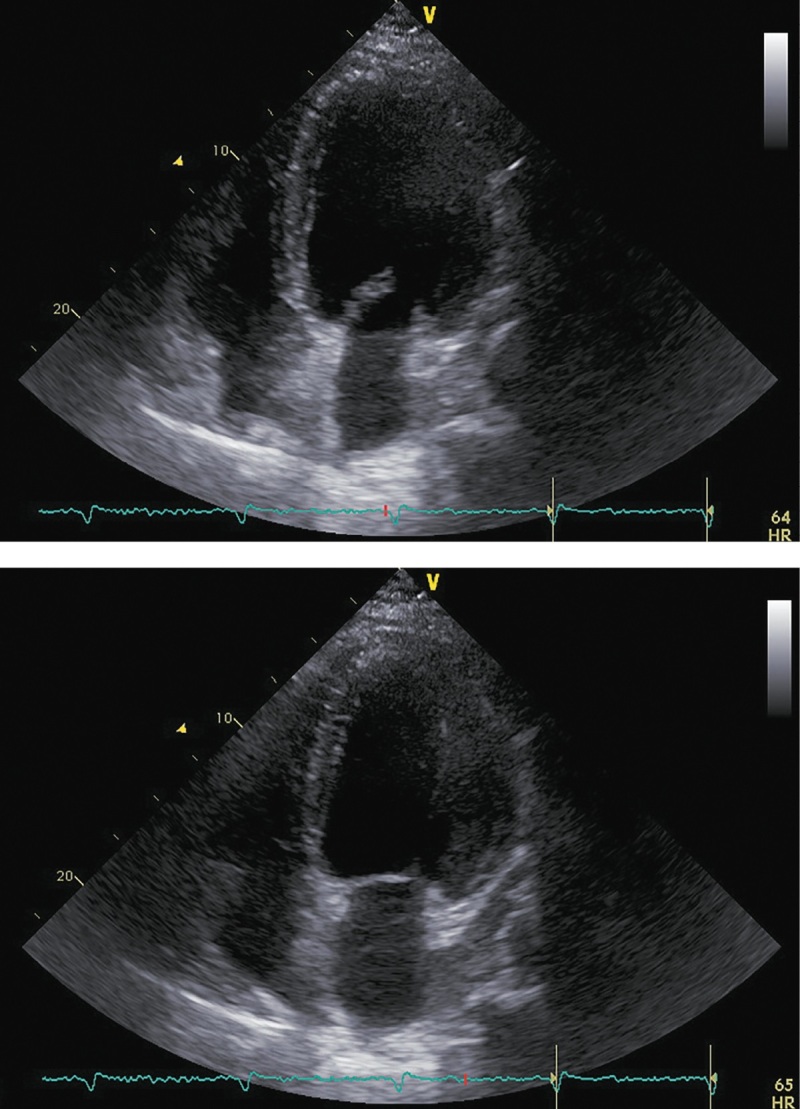
Рисунок 4. ЭХОКГ, 1 месяц до аденомэктомии. Примечание: верхушечная 4-камерная позиция (сверху представлен кадр в диастолу, снизу — в систолу).

**Table:** Таблица 2. ЭХОКГ-показатели, 1 месяц до аденомэктомии

Показатель	Значение/индекс
Объем левого предсердия	135 мл/54 мл/м²
Передне-задний размер левого желудочка	7,20 см/2,89 см/м²
Конечно-диастолический объем левого желудочка	369 мл/148 мл/м²
Конечно-систолический объем левого желудочка	297 мл/119 мл/м²
Локальная кинетика левого желудочка	диффузный гипокинез
Фракция выброса левого желудочка (усреднение по 4- и 2-камерным верхушечным позициям)	19%
Диастолическая функция левого желудочка	градация III, рестриктивный тип
Конечно-диастолическая площадь правого желудочка	27 см²/10,8 см²/м²
Конечно-систолическая площадь правого желудочка	13,5 см²/5,4 см/м²
Фракция систолического уменьшения площади	50%
Расчетное систолическое давление в легочной артерии	50 мм рт.ст.

В ноябре 2017 г. пациент госпитализирован повторно для проведения нейрохирургического лечения. Пациент указал, что принимал предписанную терапию в полном объеме. Каких-либо нежелательных лекарственных реакций не выявлено. На фоне лечения симптомы ХСН заметно регрессировали, толерантность к физическим нагрузкам возросла до уровня II ф.к. по NYHA. Физикальной симптоматики легочного застоя и увеличения печени не отмечалось. При контрольной ЭХОКГ (рис. 5; табл. 3) выявлены уменьшение размеров и объемов левого желудочка, значительное увеличение ФВ ЛЖ (до 43%) и снижение класса диастолической дисфункции ЛЖ.

**Figure:**
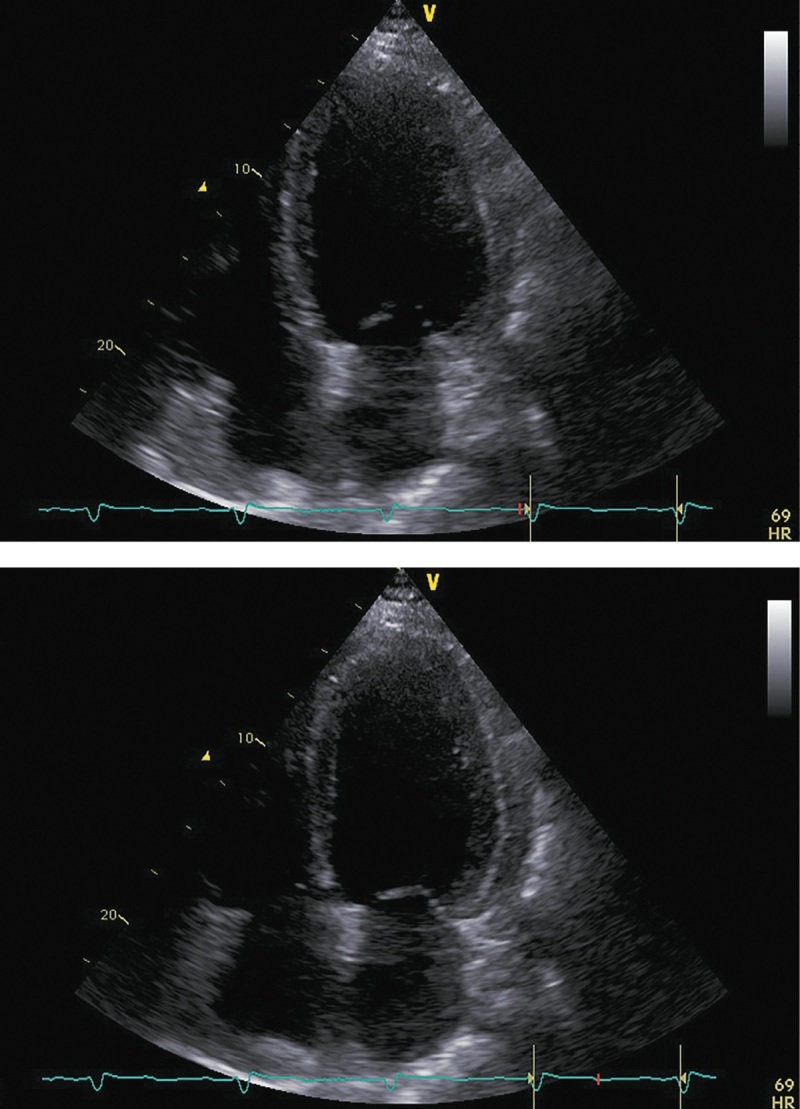
Рисунок 5. ЭХОКГ перед аденомэктомией. Примечание: верхушечная 4-камерная позиция (сверху представлен кадр в диастолу, снизу — в систолу).

**Table:** Таблица 3. ЭХОКГ-показатели перед аденомэктомией

Показатель	Значение/индекс
Объем левого предсердия	100 мл/54 мл/м²
Передне-задний размер левого желудочка	7,20 см/2,89 см/м²
Конечно-диастолический объем левого желудочка	285 мл/117 мл/м²
Конечно-систолический объем левого желудочка	162 мл/67 мл/м²
Локальная кинетика левого желудочка	умеренный диффузный гипокинез
Фракция выброса левого желудочка (усреднение по 4- и 2-камерным верхушечным позициям)	43%
Диастолическая функция левого желудочка	градация II, псевдо-нормальный тип
Конечно-диастолическая площадь правого желудочка	25,0 см²/10,0 см²/м²
Конечно-систолическая площадь правого желудочка	12,0 см²/4,8 см/м²
Фракция систолического уменьшения площади	52%
Расчетное систолическое давление в легочной артерии	43 мм рт.ст.

После стабилизации кардиального статуса пациента была проведена эндоскопическая трансназальная транссфеноидальная аденомэктомия. Течение постоперационного периода — без каких-либо кардиальных осложнений. Больной через сутки переведен из ОРИТ в хирургическое отделение.

## ИСХОДЫ И КОНТРОЛЬНЫЕ НАБЛЮДЕНИЯ

По результатам постоперационных гормональных тестов отмечены снижение уровня АКТГ и кортизола (табл. 1) и развитие вторичной надпочечниковой недостаточности, что является показателем эффективности/радикальности проведенного вмешательства.

В послеоперационном периоде инициирована заместительная терапия (рис. 1).

При последующих госпитализациях для динамического наблюдения (май 2018 г.; декабрь 2018 г.; декабрь 2019 г.) состояние пациента соответствовало стабильной клинической ремиссии. Заместительная терапия была отменена в декабре 2018 г., в дальнейшем показатели секреции кортизола находились в пределах референсных значений (табл. 1). Толерантность к физическим нагрузкам оставалась на уровне II ф.к. по NYHA. Физикальная симптоматика, характерная для ХСН, отсутствовала. При проведении контрольных ЭХОКГ-исследований отмечалось дальнейшее уменьшение размеров и объемов левого желудочка со стабилизацией ФВ ЛЖ на субнормальном уровне (рис. 6, 7, 8; табл. 4, 5, 6 соответственно). В связи с сохраняющейся АГ поликомпонентная гипотензивная терапия была продолжена в том же объеме с добавлением к ней альфа-адреноблокатора (доксазозин 2 мг два раза в сутки). СД контролировался метформином с достижением целевого уровня гликемии и гликированного гемоглобина. Уровень креатинина оставался стабильным, значимой динамики СКФ обнаружено не было (табл. 7).

**Figure:**
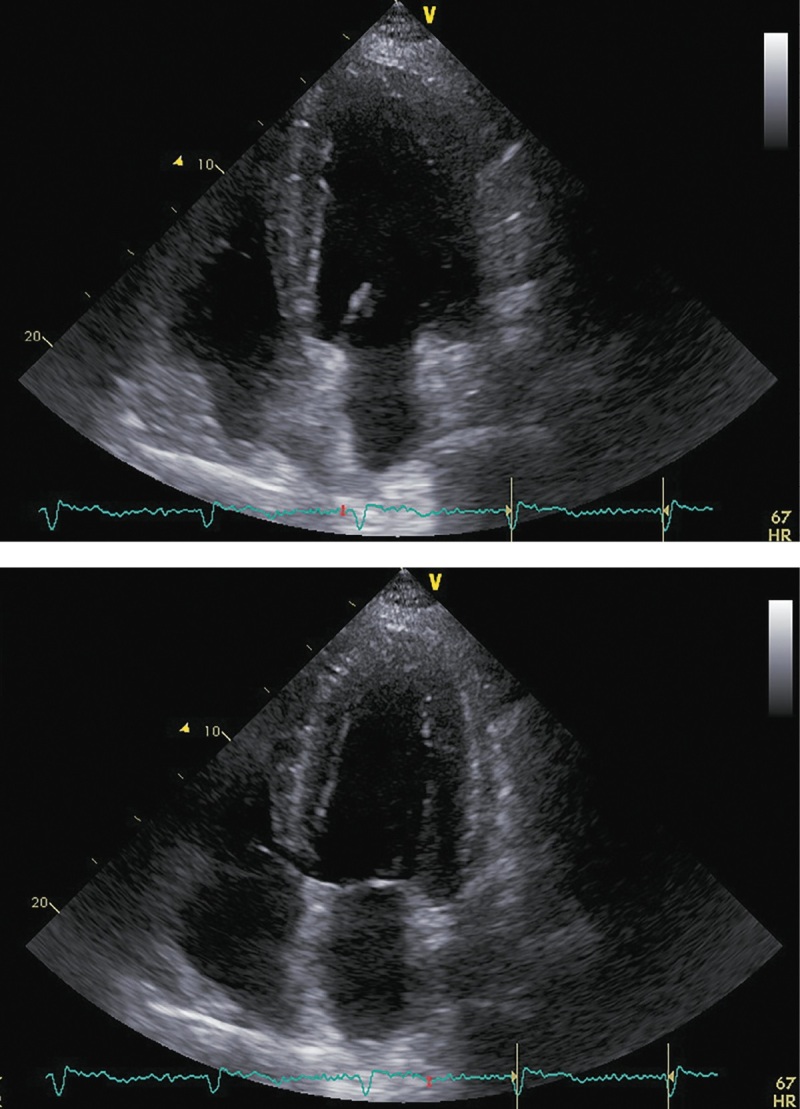
Рисунок 6. ЭХОКГ через 6 месяцев после аденомэктомии. Примечание: верхушечная 4-камерная позиция (сверху представлен кадр в диастолу, снизу — в систолу).

**Figure:**
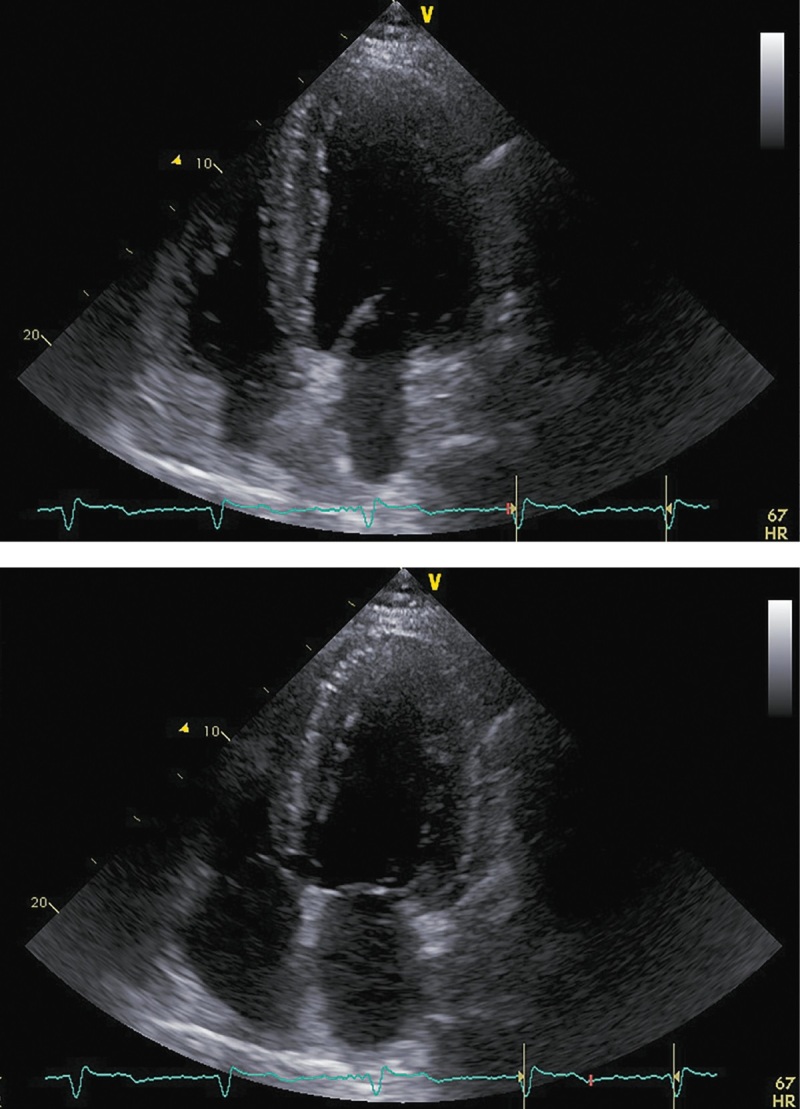
Рисунок 7. ЭХОКГ через 12 месяцев после аденомэктомии. Примечание: верхушечная 4-камерная позиция (сверху представлен кадр в диастолу, справа — в систолу.

**Figure:**
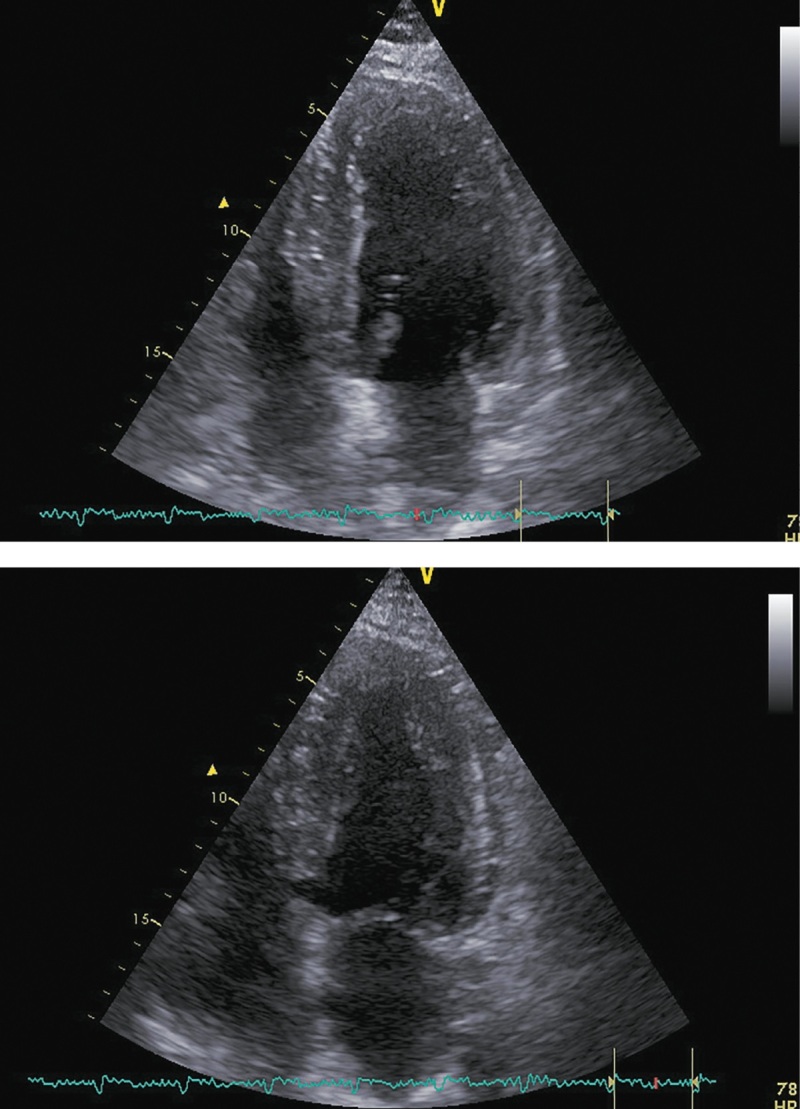
Рисунок 8. ЭХОКГ через 24 месяца после аденомэктомии. Примечание: верхушечная 4-камерная позиция (сверху представлен кадр в диастолу, справа — в систолу).

**Table:** Таблица 4. ЭХОКГ-показатели через 6 месяцев после аденомэктомии

Показатель	Значение/индекс
Объем левого предсердия	77 мл/33 мл/м²
Передне-задний размер левого желудочка	6,10 см/2,44 см/м²
Конечно-диастолический объем левого желудочка	211 мл/91 мл/м²
Конечно-систолический объем левого желудочка	103 мл/44 мл/м²
Локальная кинетика левого желудочка	Нормальная кинетика
Фракция выброса левого желудочка (усреднение по 4- и 2-камерным верхушечным позициям)	51%
Диастолическая функция левого желудочка	градация II, псевдо-нормальный тип
Конечно-диастолическая площадь правого желудочка	25,5 см²/10,9 см²/м²
Конечно-систолическая площадь правого желудочка	14,8 см²/6,4 см/м²
Фракция систолического уменьшения площади	41%
Расчетное систолическое давление в легочной артерии	41 мм рт.ст.

**Table:** Таблица 5. ЭХОКГ-показатели через 12 месяцев после аденомэктомии

Показатель	Значение/индекс
Объем левого предсердия	81 мл/34 мл/м²
Передне-задний размер левого желудочка	5,2 см/2,08 см/м²
Конечно-диастолический объем левого желудочка	174 мл/74 мл/м²
Конечно-систолический объем левого желудочка	90 мл/38 мл/м²
Локальная кинетика левого желудочка	Нормальная кинетика
Фракция выброса левого желудочка (усреднение по 4- и 2-камерным верхушечным позициям)	49%
Диастолическая функция левого желудочка	градация I, нарушения релаксации
Конечно-диастолическая площадь правого желудочка	22,9 см²/9,8 см²/м²
Конечно-систолическая площадь правого желудочка	11,2 см²/4,8 см/м²
Фракция систолического уменьшения площади	51%
Расчетное систолическое давление в легочной артерии	48 мм рт.ст.

**Table:** Таблица 6. ЭХОКГ-показатели через 24 месяца после аденомэктомии

Показатель	Значение/индекс
Объем левого предсердия	97 мл/41 мл/м²
Передне-задний размер левого желудочка	5,2 см/2,19 см/м²
Конечно-диастолический объем левого желудочка	170 мл/71 мл/м²
Конечно-систолический объем левого желудочка	77 мл/32 мл/м²
Локальная кинетика левого желудочка	Нормальная кинетика
Фракция выброса левого желудочка (усреднение по 4- и 2-камерным верхушечным позициям)	55%
Диастолическая функция левого желудочка	градация I, нарушения релаксации
Конечно-диастолическая площадь правого желудочка	20,9 см²/8,8 см²/м²
Конечно-систолическая площадь правого желудочка	11,2 см²/4,6 см/м²
Фракция систолического уменьшения площади	47%
Расчетное систолическое давление в легочной артерии	39 мм рт.ст.

**Table:** Таблица 7. Динамика СКФ (по формуле CKD-EPI), HbA1c и ИМТ

Дата исследования	СКФ (мл/мин/1,73 м²)	HbA1c (%)	HbA1c (ммоль/моль)	ИМТ (кг/м²)
Дооперационные показатели
Июль 2017	34,7	7,5	58,5	44,8
Октябрь 2017	75,8	6,7	49,7	-
Ноябрь 2017	81,1	-	-	39,8
Послеоперационные показатели
Май 2018	97,7	-	-	36,4
Декабрь 2018	56,9	-	-	36,8
Декабрь 2019	103,7	7,1	54,1	39,3

## ОБСУЖДЕНИЕ

Одной из основных причин смерти пациентов с ЭГ являются сердечно-сосудистые заболевания [6]. БИК ассоциируется с высоким риском инфаркта миокарда/ишемического инсульта [7] и характеризуется высокой распространенностью субклинических атеросклеротических поражений сонных и коронарных артерий [8–11]. Также у пациентов с ЭГ описаны редкие случаи поражения миокарда c морфо-функциональным фенотипом ДКМП [1][2].

Оптимальное лечение таких пациентов представляет серьезную клиническую проблему. Экспертные рекомендации по ведению таких пациентов отсутствуют. Более того, редкость патологии делает практически невозможным проведение РКИ, которые позволили бы оценить эффективность тех или иных вмешательств с точки зрения доказательной медицины. К настоящему времени мы обнаружили лишь два нарративных специализированных обзора, в которых представлено краткое описание случаев ДКМП у пациентов с ЭГ [1][2].

Проблемы ведения пациентов с кортизол-индуцированными ДКМП определяются не только редкостью патологии, но и сложностью диагностики. В нашем случае появление симптомов ХСН предшествовало манифестации типичной симптоматики ЭГ. Несмотря на развитие характерных для ЭГ симптомов, диагноз заподозрен и затем подтвержден только в рамках исследования перед имплантацией устройства для ресинхронизирующей терапии, через 3 года с момента верификации диагноза ДКМП.

Серьезной проблемой также стал выбор оптимальной тактики ведения пациента. Наличие выраженной СН с низкой ФВ ЛЖ не позволяло выполнить оперативное вмешательство в связи с высоким риском неблагоприятных периоперационных кардиальных событий. Была выбрана тактика отсроченного оперативного вмешательства с попыткой медикаментозной стабилизации состояния с помощью ИАПФ, бетаАБ, диуретиков, ингибиторов стероидогенеза. Стабилизировать состояние пациента удалось только после назначения максимально переносимых доз ИАПФ, бетаАБ, БМКК, диуретиков и увеличения дозы ингибитора стероидогенеза кетоконазола. Исчезновение физикальных симптомов, снижение функционального класса ХСН и значительное увеличение ФВ ЛЖ позволило выполнить эндоскопическую трансназальную транссфеноидальную аденомэктомию без каких-либо осложнений.

В большинстве анализируемых литературных источников представлена сходная тактика ведения пациентов с КИДКМП, предполагающая период предоперационной фармакологической стабилизации [1][12–21] с помощью бетаАБ, ИАПФ, диуретиков, верошпирона, а также ингибиторов стероидогенеза кетоконазола или метирапона [13][14][16][17][19] и хирургическое устранение источника гиперсекреции кортизола или АКТГ.

После хирургического вмешательства и коррекции гиперкортицизма практически во всех представленных в литературе случаях отмечено не только улучшение клинической симптоматики и снижение функционального класса СН, но и обратимость КМП — уменьшение размеров/объемов ЛЖ и значительное увеличение ФВ ЛЖ [12–22]. Схожая динамика клинических симптомов и ЭХОКГ-показателей отмечена и в описанном нами случае. Таким образом, результаты позволяют сделать предположение о возможной обратимости морфофункциональных изменений сердца у пациентов с КИДКМП при достижении эукортицизма.

Механизмы кортизол-индуцированного ремоделирования миокарда, приводящие к формированию морфофункционального фенотипа ДКМП, представляются сложными и недостаточно изученными. К сожалению, формат публикации не позволяет предоставить детальный анализ патогенеза КИДКМП. Тем не менее анализ клинико-экспериментальных исследований позволяет выдвинуть гипотезу, что ремоделирование миокарда при болезни/синдроме Кушинга является результатом совокупного воздействия избытка глюкокортикоидов (ГК) на глюкокортикоидные (ГКР) и минералокортикоидные (МКР) рецепторы миокарда.

Морфологические исследования биоптатов миокарда пациентов с КИДКМП и экспериментальные исследования описывают гипертрофию и апоптоз кардиомиоцитов, дезорганизацию миофибрилл, протеолиз сократительных белков, а также интерстициальный и периваскулярный фиброз миокарда [22–30].

Анализ экспериментальных исследований позволяет предположить, что ГК регулируют метаболизм тяжелых цепей миозина в зависимости от концентрации и времени воздействия. При низких концентрациях и коротких сроках воздействия ГК преобладает синтез миозина, при увеличении концентраций и продолжительности воздействия начинает преобладать их лизис [22][31–33]. Основным механизмом лизиса тяжелых цепей миозина может быть кортизол-индуцированная активация убиквитин-протеасомной системы кардиомицоцитов. Под воздействием кортизола происходит повышение экспрессии генов убиквитин-лигаз Atrogin1/MAFbx/MuRF1 [22][33–38] и С3 субъединицы протеасом [36] в кардиомиоцитах.

Особенностью кардиомиоцитов является отсутствие или низкая экспрессия гена 11β-гидроксистероиддегидрогеназы типа 2 (11β-HSD2), что позволяет ГК связываться как с ГКР, так и с МКР. ГК проявляют двойственное действие при взаимодействии с МКР: в нормальных условиях они оказывают ингибирующий эффект; в патологических, особенно в условиях оксидативного стресса, кортизол начинает действовать как агонист МКР. При оксидативном стрессе плотность ГКР снижается и реализуются минералокортикоидные эффекты ГК [41][42], что приводит к структурно-функциональной дезорганизации митохондрий, снижению синтеза АТФ и дальнейшей активации оксидативного стресса [43].

Кроме того, избыток ГК вызывает структурную дезорганизацию и дисфункцию митохондрий, вероятно, через активацию ГК-рецепторов митохондрий/ГК-отвечающего элемента или AngII-зависимого сигнального пути. Избыток ГК снижает экспрессию генов, кодирующих структурные компоненты и ферменты митохондрий, нарушает синтез АТФ и индуцирует оксидативный стресс [25][28][39][40].

Развитие фиброза миокарда рассматривается как следствие минералкортикоидных эффектов ГК или ГК-потенцированной активации AngII-сигнального пути [25][30][44].

Таким образом, патогенез включает 3 основных звена: 1) убиквитин-протеасомная деструкцией сократительных элементов кардиомиоцитов, 2) митохондриальная дисфункция, а также 3) фиброз миокарда.

## ЗАКЛЮЧЕНИЕ

Избыток кортизола может приводить к поражению сердца с клинико-морфологическим фенотипом ДКМП. Развитие симптомов ХСН и поражение миокарда с морфофункциональным фенотипом ДКМП могут быть доминирующими клиническими манифестациями и предшествовать появлению других клинических симптомов, характерных для ЭГ. Для безопасного хирургического удаления источника гиперсекреции АКТГ/кортизола у пациентов с КИДКМП предпочтительна тактика отсроченного вмешательства с попыткой фармакологической стабилизации состояния пациента с применением стандартной терапии ХСН и ингибиторов стероидогенеза. Поражение миокарда является обратимым при достижении эукортицизма.

Учитывая редкость КИДКПМ и отсутствие возможности проведения РКИ для определения оптимальной тактики ведения пациентов, представляется целесообразным проведение обзора по методологии scoping-review с анализом всех представленных в литературе случаев. Кроме того, целесообразно проведение отдельного обзорного исследования для анализа молекулярных механизмов ГК-индуцированного ремоделирования сердца.

## ДОПОЛНИТЕЛЬНАЯ ИНФОРМАЦИЯ

Источники финансирования. Работа выполнена по инициативе авторов без привлечения финансирования.

Конфликт интересов. Авторы декларируют отсутствие явных и потенциальных конфликтов интересов, связанных с содержанием настоящей статьи.

Участие авторов. Все авторы одобрили финальную версию статьи перед публикацией, выразили согласие нести ответственность за все аспекты работы, подразумевающую надлежащее изучение и решение вопросов, связанных с точностью или добросовестностью любой части работы.
